# Transesophageal vs. intracardiac echocardiographic screening in patients undergoing atrial fibrillation ablation with uninterrupted rivaroxaban

**DOI:** 10.1186/s12872-017-0607-1

**Published:** 2017-06-29

**Authors:** A. Tsyganov, A. Shapieva, V. Sandrikov, S. Fedulova, S. Mironovich, A. Dzeranova, E. Lyan

**Affiliations:** 1 Cardiac Electrophysiology Department, Petrovsky National Research Centre of Surgery, Abrikosovsky per. 2, Moscow, 119991 Russia; 2 Department of Clinical Physiology, Radiology and Diagnostic Imaging, Petrovsky National Research Centre of Surgery, Abrikosovsky per. 2, Moscow, Russia; 3 Cardiac Electrophysiology Department, Mechnikov North-West State Medical University, Kirochnaya ul. 41, Saint Petersburg, 191015 Russia

**Keywords:** NOAC, Transesophageal echocardiography, ICE, Atrial fibrillation, Catheter ablation

## Abstract

**Background:**

Patients with atrial fibrillation (AF) routinely undergo different imaging modalities for the evaluation of the left atrial (LA) appendage to rule out thrombus prior to the AF ablation procedure. Recently, uninterrupted novel oral anticoagulants were introduced for patients undergoing atrial fibrillation (AF) ablation to minimize the peri-procedural thromboembolism risk. We performed a retrospective analysis to evaluate the safety of uninterrupted rivaroxaban and whether transesophageal (TEE) or intracardiac echocardiography (ICE) is necessary for patients undergoing AF ablation.

**Methods:**

Data from 332 consecutive patients (42% females, aged 64 ± 11 years) with AF undergoing either TEE (*n* = 115) prior to catheter ablation or ICE (*n* = 217) for the detection of LA thrombus were analyzed. All patients were on uninterrupted rivaroxaban during, and for at least, 4 weeks before the procedure. Heparin bolus was administered in all patients before transseptal puncture to maintain a target activated clotting time of >350 s.

**Results:**

A total of 277 patients (80.4%) had paroxysmal AF. The average CHA2DS2­VASc score was 2.11 ± 0.91 in the TEE group and 2.46 ± 0.61 in the ICE group. The CHA2DS2­VASc score was ≥2 in 64 (55.7%) and 214 (98.6%) patients in the TEE and ICE groups, respectively. The left atrial appendage was adequately visualized in all cases. None of the patients have an identifiable LA thrombus either in the TEE group or the ICE group. One (0.3%) thromboembolic periprocedural stroke occurred in a patient with long-standing persistent AF in the TEE group.

**Conclusions:**

This study illustrates that performing AF ablation with ICE guidance on uninterrupted rivaroxaban for at least 4 weeks even without TEE is feasible and safe.

## Background

Radiofrequency catheter isolation of pulmonary veins has evolved into a cornerstone strategy in the treatment of symptomatic atrial fibrillation (AF) [1]. The procedural complexity and its operator dependency expose patients to a significant number of potential complications [2]. Periprocedural cerebrovascular events are a recognized complication [3]. The presence of left atrial (LA) thrombi is considered to be an absolute contraindication for interventions in the LA, because of increased risk of clot dislodgement and subsequent thromboembolic event with catheter manipulation during the ablation procedure. Many strategies have been developed to reduce the incidence of periprocedural stroke in patients undergoing ablation for AF. These include routine peri-procedural anticoagulation as well as transesophageal echocardiography (TEE) immediately prior to the procedure to rule out the presence of LA thrombus [4–7].

The main purpose of our study was to evaluate the safety of uninterrupted novel oral anticoagulants (NOAC) in patients presenting for the AF ablation. Secondly, we identified the incidence of LA thrombi in patients with uninterrupted NOAC despite 4 weeks of therapeutic anticoagulation and determined whether pre-procedural TEE should be recommended in all patients.

## Methods

### Patient selection

We retrospectively analyzed 332 consecutive patients treated with NOAC who were referred to two centers between September 2013 and October 2016. All patients had documented symptomatic AF and underwent radiofrequency catheter ablation for AF with uninterrupted rivaroxaban. Baseline demographic and clinical characteristics, including age, gender, BMI, hypertension, diabetes mellitus, coronary artery disease, and congestive heart failure, were recorded for all patients. Data related to the diagnosis of AF, including AF pattern, oral anticoagulation, and previous interventions, was also recorded. Risk stratification for thromboembolism and bleeding was performed based on the CHA2DS2-VASc and HAS-BLED score, respectively. Creatinine clearance was estimated by Cockcroft-Gault eq. A 2D transthoracic echocardiogram was carried out on all patients to estimate left ventricular ejection fraction and any structural heart diseases.

This study was approved by the Institutional Review Board of Petrovsky National Research Centre of Surgery (IRB No. 130/1).

### Transesophageal echocardiography

TEE was performed (iE33, Philips Medical Systems, MA, US) and interpreted by an experienced echocardiographer for the presence or absence of the LA thrombus. A thrombus was considered to be present if a mass detected in the LA appendage or other sites appeared to be distinct from the underlying endocardium in more than one imaging plane and was not caused by pectinate muscles.

### Intracardiac echocardiography

**Fig. 1:**
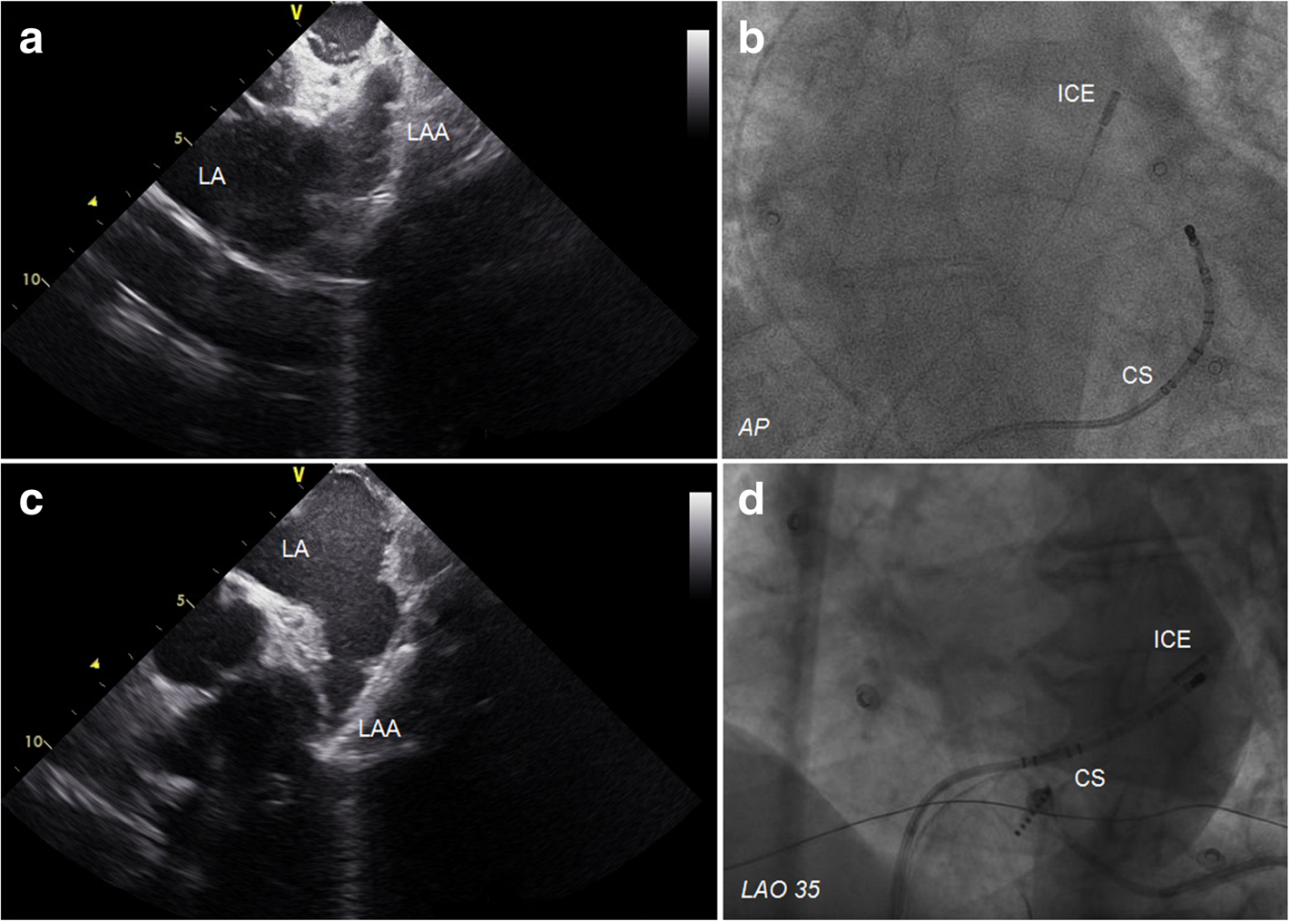
The upper row is demonstrating an example of the intracardiac echocardiographic image of the left atrial appendage from the pulmonary artery (**a**), and a fluoroscopic view of the intracardiac echocardiography probe in the pulmonary artery (**b**). The lower row is demonstrating an example of the intracardiac echocardiographic image of the left atrial appendage from the coronary sinus (**c**), and a fluoroscopic view of the intracardiac echocardiography probe in the coronary sinus (**d**)

ICE was performed by using a 10F AcuNav phased-array probe (Siemens AG, Germany) connected to a Vivid *i* system (General Electric, IL, US), and advanced through the left femoral vein. The catheter was initially positioned in the right atrium and then was rotated to visualize whole left atrium including the appendage. It was then curved and advanced into the right ventricle next to the pulmonic artery for a better visualization of the left atrial appendage. In some cases, the ICE probe was advanced into the coronary sinus, and LA appendage imaging was performed (Fig 1). Moreover, ICE was used during the procedure for the identification of procedure-related complications.

### Procedure protocol

All patients received 20 mg of rivaroxaban 4 weeks prior to the procedure without discontinuation in the intra-procedural period. The LAA visualization approach was chosen by the operator, thus neither in the TEE nor the ICE group, we did not use both modalities. All ablation procedures were performed under deep sedation. A bolus of heparin (100 IU/L) was administered immediately after vascular access before double transseptal puncture. Anticoagulation therapy was continued throughout the procedure with a targeted activated clotting time (ACT) above 350 s. The ACT was repeated every 20–30 min, and additional bolus was administrated to maintain target ACT. Transseptal sheaths were continuously flushed with heparinized saline (2000 IU/L) 180 ml per hour. A 3D map of the LA geometry was created using the Carto 3 navigation system (Biosense Webster Inc., CA, US). Continuous point-by-point ablation using a 3.5 mm irrigated open-tip catheter (ThermoCool SmartTouch D-F curve, Biosense Webster Inc., CA, US) was used to create a pulmonary vein antrum lesion set in all patients. For patients with long-standing persistent AF were performed additional linear ablation across the LA roof, in the mitral valve isthmus or LA anteroseptal region, and in the cavotricuspid isthmus. The cavotricuspid line was applied in patients with previously reported or observed typical atrial flutter. In the case of spontaneous induction of atypical atrial flutter, an activation map had been constructed, and ablation of the zone of interest was done. A deflectable circular mapping catheter (Lasso 2515, Biosense Webster inc., CA, US) was used for verification of the pulmonary veins isolation and line conduction block. At the end of the procedure, if pulmonary vein isolation (PVI) alone or plus linear did not achieve sinus rhythm, electrical cardioversion was performed.

All complications within the first 48 h after the ablation procedure were included in this study. All patients were recommended to continue rivaroxaban for at least 3 months or long-life after discharge if the CHA2DS2-VASc score was 0 or ≥1, respectively.

### Data analysis

Continuous variables were expressed as the mean ± standard deviation. Categorical variables were expressed as absolute numbers and percentages. Differences between means among groups were compared using the Student *t* test for continuous variables and with the nonparametric χ2 test or Fisher exact test when appropriate for categorical variables. A *p* value less than 0.05 was considered significant. SPSS software version 20.0 (IBM, NY, US) was used for statistical analysis.

## Results

### Patient characteristics

A total of 115 patients (37 females, mean age 57.4 ± 11.5 years) underwent TEE prior to catheter ablation, and 217 patients (103 female, mean age 67.6 ± 8.3 years) underwent ICE during the procedure without prior TEE. Patients in the ICE group tended to be older, having higher BMI and LA size, and more often suffer from hypertension and diabetes mellitus, while the incidence of coronary artery disease was lower. The average CHA2DS2­VASc tended to be higher in the ICE group, but HAS-BLED scores were similar in both groups. However, the CHA2DS2­VASc score was ≥2 only in 64 (55.7%) patients in the TEE group versus 214 (98.6%) patients in the ICE group. Two hundred and sixty-seven patients (80.4%) had paroxysmal AF, 56 patients (16.9%) had persistent AF, and 9 patients (2.7%) had long-persistent AF. Thirty-eight patients underwent a redo procedure after the previous PVI. Three patients had a concomitant atypical atrial flutter before the procedure. In one patient, atypical atrial flutter was observed during the procedure.

The left atrial appendage was adequately visualized in all cases. None of the patients have an identifiable LA thrombus either in the TEE group or ICE group.

Baseline characteristics of the study population are summarized in Table 1.

**Table 1:** Demographic patient data

	TEE group	ICE group	*p-*value
Paroxysmal	71 (61,7%)	196 (90,3%)	<0.001
Persistent	38 (33%)	18 (8,3%)	<0.001
Long-standing persistent	6 (5,3%)	3 (1,4%)	0.042
Age, years	57.4 ± 11.5	67.6 ± 8.3	<0.001
BMI	28.6 ± 4.5	29.7 ± 4.6	0.044
Coronary artery disease	27 (23.5%)	1 (0.4%)	<0.001
Hypertension	78 (67.8%)	210 (96.8%)	<0.001
Diabetes mellitus	11 (9.6%)	65 (29.9%)	<0.001
Congestive heart failure	16 (13.9%)	22 (10.1%)	0.312
Previous stroke or TIA	1 (0.8%)	0 (0%)	0.951
Left atrial size, mm	41.9 ± 6.2	43.4 ± 3.7	0.006
LV EF, %	58.9 ± 8.3	60.2 ± 7.7	0.194
Creatinine clearance, ml/min	108.81 ± 32.76	112.06 ± 24.05	0.092
CHA_2_DS_2_-VASc score	2.09 ± 0.91	2.46 ± 0.61	0.048
0	23 (20%)	1 (0.4%)	<0.001
1	28 (24.3%)	2 (0.9%)	<0.001
≥2	64 (55.7%)	214 (98.6%)	<0.001
HAS-BLED	0.85 ± 0.89	0.96 ± 0.84	0.071

Data are presented as mean ± SD

*AF* atrial fibrillation, *BMI* body mass index, *TIA* transient ischemic attack, *TEE* transesophageal echocardiogram, *ICE* intracardiac echocardiography, *LV EF* left ventricular ejection fraction

### Clinical outcomes

PVI was achieved in all patients. In nine patients with long-standing, persistent AF, linear ablation was performed. A total of 37 patients underwent the cavotricuspid isthmus ablation. All patients with atypical atrial flutter were successfully mapped and ablated; of which, two had peri-mitral, one had peri-venous, and the other one had right atrial upper loop re-entry flutter. The mean procedure time was 101.5 ± 15.1 in the TEE group and 110.8 + 12.4 min in the ICE groups (*p* = 0.78).

One (0.3%) thromboembolic event occurred in a patient on uninterrupted 20 mg daily rivaroxaban. The patient had the persistent type of atypical atrial flutter after previous PVI and cavotricuspid isthmus ablation. His CHA2DS2-VASc score was equal to 2. He underwent the repeat procedure for PVI and linear ablation for right atrial upper loop re-entry flutter. The sinus rhythm was restored during linear ablation in the right atrium between the superior and inferior vena cava. The day after the procedure, the patient had an acute stroke with right temporal hemianopia. Magnetic resonance imaging revealed an acute infarction in the left parietal lobe.

A total of 6 (1.8%) patients had bleeding complications. Two (0.6%) major bleeding complications were pericardial effusion with cardiac tamponade, managed by percutaneous pericardial drainage with no sequelae. Four patients (1.2%) who had minor bleeding complications suffered a groin hematoma. No patient with groin hematoma required intervention.

## Discussion

Periprocedural screening of intracardiac thrombi and optimal anticoagulation strategy plays an important role in the prevention of thromboembolic complications associated with AF ablation. NOAC were developed as an alternative to vitamin K antagonist for prevention of thromboembolism in patients with AF. In the RELY study, the rate of LA thrombus detection among patients undergoing TEE before cardioversion was 1.2% and 1.8% for patients on dabigatran 150 mg and 110 mg, respectively. However, continuous treatment with NOAC for >3 weeks before cardioversion was less than 80%. TEE data were available in 86 patients on apixaban in the ARISTOTLE study, and none of them had LA thrombus. In the ROCKET AF study, no TEE data were collected to assess rates of LA thrombus among patients on rivaroxaban undergoing cardioversions or catheter ablation of AF [8–10]. In recent studies, the use of NOAC has been shown to be safe and effective in preventing bleeding and thromboembolic complications during uninterrupted periprocedural anticoagulation for AF ablation procedure [11–13].

At present TEE is considered the gold standard in detecting LA thrombi, with a high degree of sensitivity and specificity complying 97% and 100%, respectively [14]. Although an invaluable diagnostic tool, TEE is also a relatively invasive procedure with inherent risk. Major TEE-related complication rates range from 0.2% to 0.5%. Mechanical injury to the gastrointestinal tract can lead to life-threatening complications. The risk of significant gastrointestinal bleeding is estimated between 0.02% and 1.0% [15, 16]. Moreover, TEE may be associated with increased rates of false positive results, associated with artifacts, pectinate muscles, or “smoke” in the LA appendage [17]. However, several studies have shown that AF ablation can be performed without TEE screening when patients are anticoagulated with NOAC [12, 18]. In a prospective multicenter trial, Di Biase et al. showed that AF ablation procedure in patients on uninterrupted NOAC is feasible and safe without TEE, including patients with high risk for stroke (the average CHA2DS2-VASc score was 3.0 ± 1.3) [19]. In that study, all patients underwent ablation procedure under ICE guidance. There was no case-detected thrombus in the LA. Our study, in which an uninterrupted strategy with rivaroxaban was also used, showed no thromboembolic complications in the ICE group, thus confirming the conclusion of Di Biase’s study. However, in a recent study, Frenkel et al. showed 3.4% of the LA thrombosis in the uninterrupted NOAC (rivaroxaban and apixaban) cohort were screened with TEE [20].

In a Task Force survey, approximately 50% of members performed TEE in all patients undergoing AF ablation, regardless of the stroke risk [21]. In many electrophysiology laboratories, ICE is used to assist in transseptal puncture and catheter navigation. Moreover, it can also be used for LA imaging when TEE is unequivocal or impossible to perform [22]. In the Action-Ice I Study, Baran et al. showed that ICE could be used safely and effectively for the evaluation of the LA appendage in patients undergoing AF ablation [23]. ICE allows proper visualization of the LA appendage in 85% to 88% of all cases. In most cases, the standard right atrial view does not provide adequate, consistent visualization of the LA appendage. The ICE catheter, when placed in locations that are more proximate to the LA appendage, such as the pulmonary artery or the coronary sinus, improves sensitivity and specificity in detecting the LA appendage thrombus [23, 24]. To the best our knowledge, the likelihood of obtaining satisfactory LA appendage visualization in different imaging planes by an experienced electrophysiologist is nearly 100%. In this study, we did not estimate other thrombogenic milieus, such as a spontaneous echo contrast or LA appendage flow velocity, for two reasons. First, it did not influence the decision to perform the ablation procedure, and there is no consensus that any TEE variable other than the LA thrombus is associated with the occurrence of periprocedural thromboembolic events [25].

The risk of the LA thrombi in patients who are fully anticoagulated with NOAC is very low, but it still exists especially in high-risk patients with CHA_2_DS_2_-VASc score ≥ 2. Furthermore, despite periprocedural anticoagulation and TEE or ICE, thromboembolic events can happen in AF patients. In our series, only one patient from the TEE group suffered from a periprocedural stroke, and unfortunately, the incidence and clinical predictors of such complication in patients undergoing AF ablation are not fully understood. Finally, our study adds evidence to the periprocedural management field of AF ablation procedure, illustrating the safety of this procedure in high-risk patients who have been on uninterrupted NOAC for at least four weeks. Use of ICE allows dismissing TEE, as well as avoiding a small, but existing risk of thromboembolic complications associated with AF ablation.

### Limitations

The major limitation of our study is its retrospective nature. Thus, the approach of LA visualization was neither randomized nor systematic, but instead a reflection of the choices of the operator. Only rivaroxaban was included; therefore, it is difficult to generalize for the entire class of NOAC. The majority of patients in the ICE group had paroxysmal AF, in which the stroke risk is already minimized as compared to the non-paroxysmal population, that reflects the biased allocation of study participants. Also, ablation strategy and experience could influence thromboembolic events during the procedure regardless of TEE or ICE screening. Most importantly, this study was underpowered to provide statistically rigorous results, and was thus, exploratory in nature.

## Conclusions

Our study illustrates that ICE may become a useful clinical tool for LA assessment in patients undergoing AF ablation. Moreover, AF ablation with ICE guidance on uninterrupted rivaroxaban for at least 4 weeks without TEE is feasible and safe.

## Abbreviations

ACT: Activated clotting time
AF: Atrial fibrillation
ICE: Intracardiac echocardiography
LA: Left atrium
NOAC: Novel oral anticoagulants
PVI: Pulmonary vein isolation
TEE: Transesophageal echocardiography
